# Linkage disequilibrium mapping of a breast cancer susceptibility locus near *RAI/PPP1R13L/iASPP*

**DOI:** 10.1186/1471-2350-9-56

**Published:** 2008-06-27

**Authors:** Bjørn A Nexø, Ulla Vogel, Anja Olsen, Mette Nyegaard, Zuzanna Bukowy, Eszter Rockenbauer, Xiuqing Zhang, Cemile Koca, Mette Mains, Bettina Hansen, Anne Hedemand, Anette Kjeldgaard, Magdalena J Laska, Ole Raaschou-Nielsen, Søren Cold, Kim Overvad, Anne Tjønneland, Lars Bolund, Anders D Børglum

**Affiliations:** 1 Institute of Human Genetics, University of Aarhus, DK-8000 Aarhus C, Denmark; 2 National Research Centre for the Working Environment, DK-2100 Copenhagen O, Denmark, Institute for Science, Systems and Models, University of Roskilde, DK-4000 Roskilde. and National Food Institute, Technical University of Denmark, Denmark; 3 Institute of Cancer Epidemiology, The Danish Cancer Society, DK-2100 Copenhagen O, Denmark; 4 Institute of Biochemistry and Biophysics, Polish Academy of Sciences, Warshawa, Poland; 5 Department of Forensic Genetics, Institute of Forensic Medicine, University of Copenhagen, DK-2100 Copenhagen O, Denmark; 6 Beijing Genomics Institute, Beijing, PR China; 7 Institute of Embryology and Histology, University of Silesia, Poland; 8 Odense University Hospital, DK-5000 Odensek, Denmark; 9 Department of Clinical Epidemiology, Aalborg Hospital and Aarhus University Hospital, DK-9000 Aalborg, Denmark

## Abstract

**Background:**

Previous results have suggested an association of the region of 19q13.3 with several forms of cancer. In the present study, we investigated 27 public markers within a previously identified 69 kb stretch of chromosome 19q for association with breast cancer by using linkage disequilibrium mapping. The study groups included 434 postmenopausal breast cancer cases and an identical number of individually matched controls.

**Methods and Results:**

Studying one marker at a time, we found a region spanning the gene *RAI *(alias *PPP1R13L or iASPP*) and the 5' portion of *XPD *to be associated with this cancer. The region corresponds to a haplotype block, in which there seems to be very limited recombination in the Danish population. Studying combinations of markers, we found that two to four neighboring markers gave the most consistent and strongest result. The haplotypes with strongest association with cancers were located in the gene *RAI *and just 3' to the gene. Coinciding peaks were seen in the region of *RAI *in groups of women of different age.

In a follow-up to these results we sequenced 10 cases and 10 controls in a 44 kb region spanning the peaks of association. This revealed 106 polymorphisms, many of which were not in the public databases. We tested an additional 44 of these for association with disease and found a new tandem repeat marker, called *RAI*-3'd1, located downstream of the transcribed region of *RAI*, which was more strongly associated with breast cancer than any other marker we have tested (RR = 2.44 (1.41–4.23, p = 0.0008, all cases; RR = 6.29 (1.49–26.6), p = 0.01, cases up to 55 years of age).

**Conclusion:**

We expect the marker *RAI*-3'd1 to be (part of) the cause for the association of the chromosome 19q13.3 region's association with cancer.

## Background

**Figure 1:**
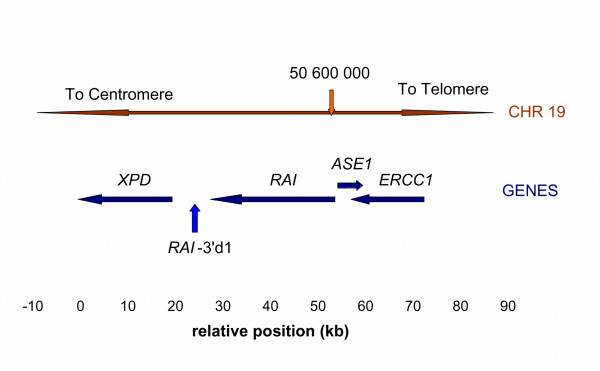
Schematic representation of the relevant gene region. The crimson arrow marks a chromosomal position. The dark blue arrows represent the 4 genes of the region. The numbers below indicate the position relative to the marker *XPD*-exon23.

The search for genetic determinants influencing the risk of cancer has gained momentum due to the very detailed maps of the human genome now available. We and others have previously shown that a region of chromosome 19q13.3 seems associated with a number of cancers, including cancer of the skin, breast, lung and brain [1-12], but not testis and colorectal cancer [13,14] Central in this chromosomal region is a 69 kb stretch, which contains two genes of importance for DNA repair, *XPD *(also known as *ERCC2*) and *ERCC1*, one gene presumably relating to apoptosis, *RAI *(also known as *iASPP *or *PPP1R13L*), and one presumably involved in ribosomal RNA transcription, *ASE1 *alias *CD3EAP *(see Figure 1). Thus three of four genes are related to the removal of damaged DNA, an important mechanism in preventing cancer.

In the present study, involving 434 breast cancer cases and an identical number of controls, we initially investigated 27 public markers within the 69 kb stretch for association with breast cancer, using linkage disequilibrium mapping based on single markers, as well as on haplotypes combining several neighboring markers. We found that the region formed two haplotype blocks, and that associations were concentrated on the gene *RAI *in one of these haplotype blocks. We resequenced 44 kb encompassing this block in 10 cases and 10 controls. Among the 106 found polymorphisms (including the 27 described above) we tested an additional 44 polymorphisms for association with disease and identified a 5-bases tandem repeat polymorphism, NT_011109.15 g.18147012dupATTTT(2_11), here called *RAI*-3'd1, which discriminates cases from controls better than any previously investigated polymorphism in this region, and which may well be the effector causing this haplotype block to associate with cancer.

## Results

This investigation deals with breast cancer in women. The cohort consisted of 24 697 Danish postmenopausal women age 50 – 64 at inclusion in 1993 – 1997. 434 of these women developed breast cancer before 2001. The cases and a set of individually matched controls (see Methods for details) were analyzed as a nested case-control study. [9]. All subjects were Caucasians.

We investigated DNA polymorphisms in a 69 kb sub-region of 19q13.3 for association with breast cancer. To improve readability each polymorphism investigated was given a trivial name in accordance with its positions relative to the nearest gene followed by a hyphen and a serial number, for instance *RAI *intron8-3 denotes the third SNP investigated in the eighths intron of *RAI*. In the relative few polymorphisms that were in/dels or repeat polymorphisms, the serial number was preceded by a d, for instance *RAI*-3'd1. The markers genotyped in this study are listed in Table 1. We initially typed the ones labelled L1 and T in the "Series" column. There was no statistical significant departure from Hardy-Weinberg equilibrium of any marker among the controls.

**Table 1:** The polymorphisms used in this study, their kind and their identification

New trivial name^1^	Systematic name^2^	Series^3^	rs#^4^	contig pos^5^	relativ position^6^
XPD-exon23	ERCC2 c.2282A>C	L1	13181	18123137	0
XPD-exon10	ERCC2 c.965G>A	L1	1799793	18135477	12340
XPD-exon6	ERCC2 c.499A>C	L1 + S	238406	18136527	13390
XPD-intron5-1	ERCC2 c.492-62T>A	S	238407	18136696	13559
XPD-intron5-2	ERCC2 c492-892A>G	S	3916809	18137426	14289
XPD-intron5-3	ERCC2 c.391+311A>G	S		18139824	16687
XPD-intron3-1	ERCC2 c.278-35G>A	L1	1799783	18140254	17117
XPD-5'1	ERCC2 c.-35-113G>C	S	3810366	18142160	19023
XPD-5'd1	ERCC2 c.-35-298_-294delGACA	L1 + S	3916791	18142345	19208
XPD-5'4	ERCC2 c.-35-583A>C	L2	3916788	18142630	19493
XPD-5'd2	ERCC2 c.-35-980_-979ins80	A	3916787	18143027	19890
XPD-5'2	ERCC2 c.-35-1958A>G	L1 + S	2097215	18144005	20868
XPD-5'3	g.18145185T>C	L1 + S	11878644	18145185	22048
RAI-3'd2	g. 18145752_18145773del(22)	A		18145768	22631
RAI-3'8	g.18142233C>A	S		18146233	23096
RAI-3'7	g.18146823C>T	L1	7252567	18146823	23686
RAI-3'd1	g.18147012ATTTT(2_11)	A	7255792	18147012	23875
RAI-3'9	g.18147126T>C	L2	2377329	18147126	23989
RAI-3'd3	g.18147199delTT	A	3047560	18147192	24055
RAI-3'10	g.18147886T>G	S + L2	10422489	18147886	24749
RAI-3'11	g.18148193C>T	L2	10426701	18148193	25056
RAI-3'4	g.18150199A>G	L1 + S	4544343	18150199	27062
RAI-exon 13-2	PPP1R13L c.*507T>G	S		18151158	28021
RAI-exon13-1	PPP1R13L c.*475T>A	T + S	6966	18151180	28043
RAI-intron12-1	PPP1R13L c.2515-68G>A	S		18151772	28635
RAI-intron12-4	PPP1R13L c.2515-330G>C	S		18152034	28897
RAI-intron12-3	PPP1R13L c.2515-467G>A	L1	10417235	18152171	29034
RAI-intron12-5	PPP1R13Lc.2515-1150G>A	L2	12876252	18152764	29627
RAI-intron12-2	PPP1R13L c.2514+505T>A	L1	8112723	18153497	30360
RAI-intron11-2	PPP1R13L c.2315-594T>G	S		18154796	31659
RAI-intron11-1	PPP1R13L c.2315-1281G>A	L1 + S	2017104	18155483	32346
RAI-intron11-2	PPP1R13L c.2248+1167G>T	S		18155871	32734
RAI-intron8-1	PPP1R13L c.1882-1425A>G	L1	1970764	18159091	35954
RAI-intron8-10	PPP1R13L c.1882-1263A>G	S		18159263	36126
RAI-intron8-9	PPP1R13L c.1882-1697A>G	L2		18160363	37226
RAI-intron8-4	PPP1R13L c.1882-2270T>G	S		18160936	37799
RAI-intron8-5	PPP1R13L c.1882-2271G>C	S		18160937	37800
RAI-intron8-11	PPP1R13l c.1881+1912G>T	S		18161433	38296
RAI-intron8-6	PPP1R13L c.1881+1514A>G	S		18161841	38704
RAI-intron8-2	PPP1R13L c. 1881+1149B>A	L1	6509210	18162206	39069
RAI-intron8-12	PPP1R13l c.1881+756C>T	L2	12986272	18162599	39462
RAI-intron8-7	PPP1R13L c 1881+452T>G	S		18162903	39766
RAI-intron8-8	PPP1R13L c.1881+389T>C	S		18162986	39849
RAI-intron8-3	PPP1R13L c.1881+385T>G	L1		18162970	39833
RAI-intron7-1	PPP1R13L c.1421-1236G>C	S		18165052	41915
RAI-intron3-2	PPP1R13L c.264-404G>A	L1	4803814	18168944	45807
RAI-intron3-3	PPP1R13L c.264-408G>A	L1	4803815	18168948	45811
RAI-intron1-5	PPP1R13L c.-21-1304insTAAG	S		18171119	47982
RAI-intron1-7	PPP1R13L c.-21-1931G>T	S	10402584	18171746	48609
RAI-intron1-10	PPP1R13Lc.-21-2062G>A	S	10401293	18171815	48678
RAI-intron1-1	PPP1R13L c.-21-2169A>G	L1 + S	4572514	18171984	48847
RAI-intron1-4	PPP1R13L c.-21-3291G>A	L2 + S	4803816	18173106	49969
RAI-intron1-8	PPP1R13L c.-21-3291A>G	S		18173107	49970
RAI-intron1-2	PPP1R13L c.-22+1148C>T	L1	2226949	18175327	52190
RAI-intron1-3	PPP1R13L c.-22+734A>G	L2	959457	18175739	52602
RAI-intron1-9	PPP1R13L c.-22+379T>C	S		18176096	52959
RAI-intron1-5	PPP1R13L c.-22+297T>C	L2 + S	4803817	18176178	53041
RAI-5'1	PPP1R13L c-54-177T>C	L1 + S	10412761	18176680	53543
ASE1-exon1	CD3EAP c.-468-21G>A	L1	967591	18178152	55015
ASE1-exon2	CD3EAP c.22+29A>C	S		18178222	55085
ASE1-exon3-4	CD3EAP c.564G>C	S		18178623	55486
ASE1-exon3-1	CD3EAP c.1264A>G	T	735482	18180220	57083
ASE1-exon3-2	CD3EAP c.1605A>G	L1 + S	762562	18180561	57424
ASE1-exon3-3	CD3EAP c.1668G>A	T + S	2336219	18180624	57487
ASE1-exon3-d1	CD3EAP c.1751TTC(5_6)	A	3212987	18180707	57570
ASE1-exon3-6	CD3EAP c.1998C>A	L1 + S	3212986	18180954	57817
ASE1-exon3-4	CD3EAP c.*1228T>C	L2	3212983	18182205	59068
ERCC1-exon4	ERCC1 c.500T>C	L1	11615	18191871	68734

1) The trivial names have been changed to conform to the new model of the *RAI *gene, which involves a major 5' extension of the gene relative to older models. Specifically, the polymorphism previously called RAI intron1 is now called RAI-intron8-1. Similarly RAI exon6 is now called RAI-exon13-1.

2) The systematic name according to

3) The series of assays to which the polymorphism belongs

L1: First series of Lightcycler assays

L2: Later series of Lightcycler assays

A: Assays on ABI3100

T: Assays on Taqman

S: Assays on Sequenom

4) The rs-number of the polymorphisms in NCBI's database dbSNP

5) Positions of the polymorphisms in the contig [GenBank: NT_011109.15]

6) Positions of the polymorphisms relative to XPD-exon23

**Figure 2:**
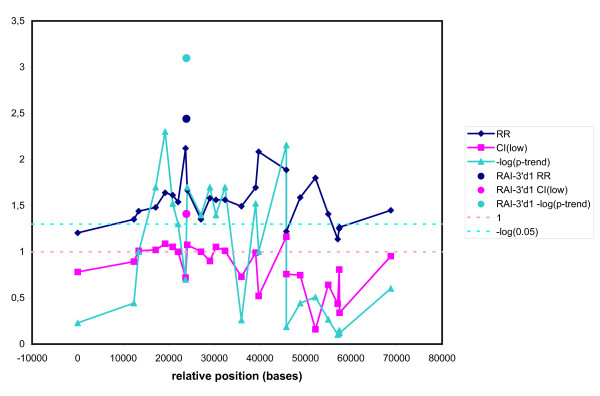
Association of single polymorphisms with breast cancer, all ages. The relative risk for cancer of the two homozygotes (Dark blue), the lower confidence limit for the relative risk(Pink), and the negative logarithm to the p-trend for association of all 3 genotypes(Light blue) of each marker with cancer is depicted against the position of the marker. The singular points represent the values for *RAI*-3'd1.

Next, we calculated the relative risks of breast cancer and the corresponding confidence limits for each marker comparing the variant homozygotes to homozygotes for the wildtype allele. However, if the calculation gave an RR value less than 1, we used the reciprocal value of the RR and the reciprocal value of the upper confidence limit instead. This analysis has the advantage that no *a priori *assumptions about the distribution of genotypes are needed. Figure 2 depicts the relative risks and the lower confidence limits of the relative risks for postmenopausal breast cancer at all ages as a function of the location of the markers. The figure also shows the logarithm of the p-values for trend of association using all 3 genotypes for each marker. Significant associations (i.e. p-values less than 0.05) of markers with cancer existed in the region from 13 000 to 40 000 bases, covering most of the gene *RAI *and the 5' region of the gene *XPD*. The singular values for the polymorphism *RAI*-3'd1 will be discussed later. The corresponding curves for women aged less than 55 showed an even higher relative risk in the same region with a peak value of RR = 6.25 (1.72 – 20), p(trend) = 0.005 (results not shown).

**Figure 3:**
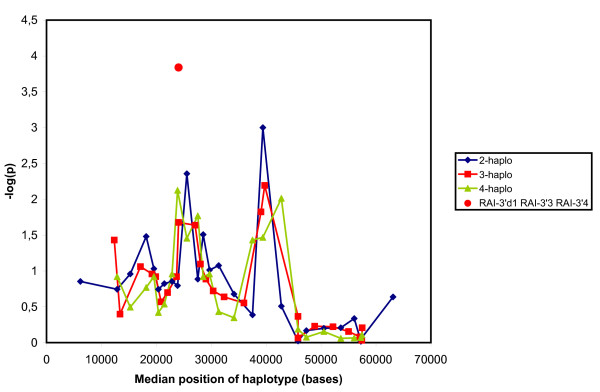
Association of sets of neighbouring SNPs with breast cancer. The overall p-value for the association of a set of markers as calculated by HelixTree is depicted against the median position of the markers. The singular point represent the value for the haplotype *RAI*-3'd1 *RAI*-3'd3 *RAI*-3'4. (Blue), haplotypes made up of 2 neighboring polymorphisms. (Red), haplotypes made up of 3 neighboring polymorphisms. (Green), haplotypes made up of 4 neighboring polymorphisms.

We also used the analytical technique known as haplotype trend regression [15], more specifically the program HelixTree, to associate sets of markers with the individual diseases. We used the entire set of controls for the analyses. This broke the matching of the data sets, but programs performing haplotype trend regression on individually matched cohort data are not available, and matching on other criteria than ethnicity and age may well be irrelevant for genetic association studies [16]. Figure 3 shows the overall distribution of p-values for sets of markers plotted against the position on the chromosome for breast cancer at all ages. The abscissa of each short haplotype was defined as the median position of the markers included. Each curve corresponds to a given size of the marker set, i.e., the number of markers in the haplotypes. The figure suggests that the association with breast cancer had two peaks. One was located at roughly 24 kb and one at 39 kb. The peaks were clearly present in curves for haplotypes of 2, 3, and 4 neighbouring markers. Larger sets were less informative, presumably because of increased degrees of freedom, but they essentially corroborated the results. When the cases were broken down into cases before and after age 55, the peak at 24 kb was present in both groups, whereas the peak at 39 kb only was present in the older group (results not shown). For comparison, the figure also contains a singular point corresponding to the best haplotype, which included the new marker *RAI*-3'd1, to be described later.

**Figure 4:**
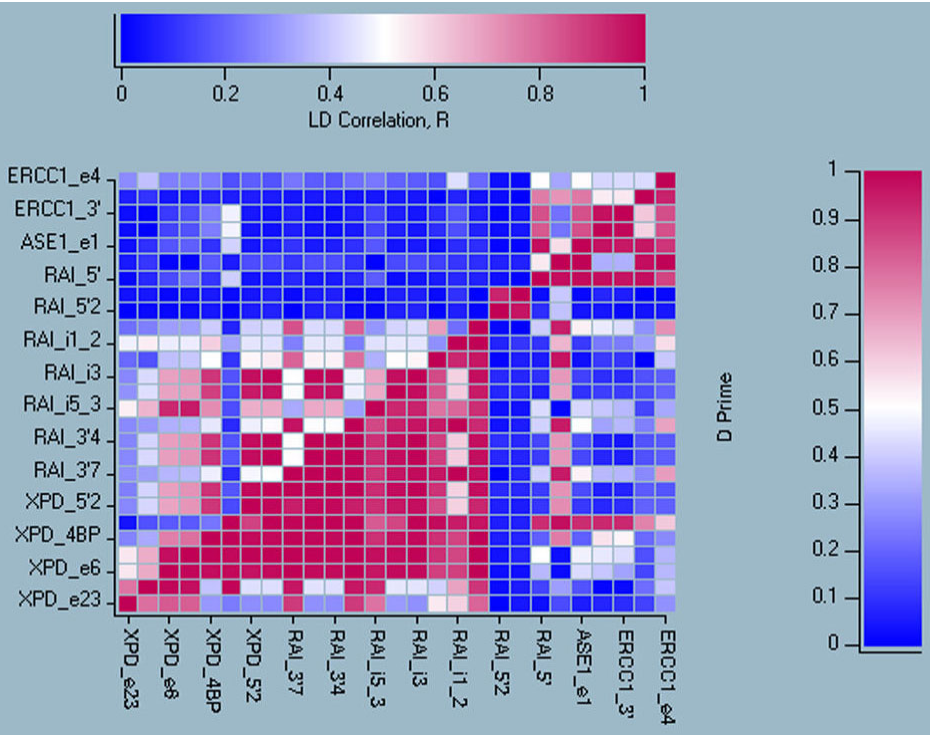
Pair-wise linkage disequilibrium between all markers in the controls. The values above the diagonal are the correlations of two polymorphisms, R. The values below the diagonal are Nowontin's normalised linkage disequilibrium, D'. The red color represent high values, blue represents low value.

Finally, we calculated the pair-wise linkage disequilibrium of all markers in the controls (Figure 4). The region consisted of two major haplotype blocks with a few interspersed markers. One haplotype block spanned 16 markers and 26 kb from *XPD *exon6 to *RAI *intron8-3, corresponding to the region with association to cancer, whereas the other haplotype block spanned 6 markers and 9 kb from *RAI *intron1-1 to *ASE1 *exon3-3.

Based on all these results we chose to sequence PCR fragments spanning the region from relative position 13 to 59 kb in breast cancer cases and controls. Initially, we selected 10 cases and 10 controls for analysis. The actual number of reliable sequences obtained varied somewhat from one part of the region to the other. Table 9 lists the 106 detected polymorphisms including their positions, their rs-numbers if assigned, and the surrounding sequences [See Additional File 1]. Essentially all previously analyzed polymorphisms were included in the list.

Based on the sequencing results, we analyzed 44 new polymorphisms using ABI3100, Lightcycler and Sequenom (designated A, L2 and S in the "Series" column of Table 1). Among the remainder 18 could not be reproduced and were probably sequencing errors rather than polymorphisms. Most of the remaining could not be assayed for due to the repetitive nature of the DNA around them. Fifteen of the polymorphisms were analyzed by two methods. The concordance of the two methods was very high. One polymorphism had a concordance of 98 percent, and the remainder had concordance rates of 99 percent or above.

A new tandem repeat polymorphism, *RAI-*3'd1 was particularly interesting. It was located close to minimum p value of the haplotype trend regression analysis and it had the potential to modify gene expression by virtue of the relative large change in the sequence. To simplify the scoring we subdivided the fragments of *RAI*-3'd1 into two groups, 6 repeats or less, called S for short, and 7 repeats or more, called L for long. The results are shown in Table 2. *RAI*-3'd1, showed a stronger association with disease than any other polymorphism we have tested (singular point in figure 2). The calculated relative risk was higher (RR = 2.44 (1.41–4.23) and the associated p-value was lower (p = 0.0008) than any we had observed before. When the effect was broken down into age brackets, *RAI*-3'd1 was marginally better (RR = 6.29 (1.49–26.6)) than other markers in the youngest age group and showed a clearer tendency in the other age groups (see Table 2).

**Table 2:** Association of breast cancer with two additional polymorphisms selected from those detected by sequencing.

**Polymorphism**	**N^**1 **^cases**	**N^**1 **^controls**	**IRR^**2 **^(95% CI**^**3**^)	**P-trend**^**4**^
***RAI*-3'd1**				
	
All ages				0.0008
LL	38	59	1	
SL	127	165	1.39 (0.81–2.38)	
SS	200	150	2.44 (1.41–4.23)	
	
<55 yr				0.01
LL	5	15	1	
SL	18	25	1.46 (0.33–6.47)	
SS	33	14	6.29 (1.49–26.61)	
	
55–60 yr				0.19
LL	12	17	1	
SL	45	63	1.72 (0.63–4.73)	
SS	61	49	2.57 (0.88–7.48)	
	
>60 yr				0.12
LL	21	27	1	
SL	64	77	1.09 (0.52–2.60)	
SS	106	87	1.73 (0.83–3.64)	

***RAI*-3'd2**				
	
All ages				0.005
LL	149	113	1	
SL	168	199	0.62 (0.44–0.88)	
SS	66	88	0.50 (0.32–0.79)	

1) Number of persons

2) Incidence rate ratio

3) Confidence limit

4) P-value for trend among the IRRs for all 3 genotypes

Another interesting polymorphism, *RAI*-3'd2, was also associated with disease, albeit to a lesser extent. Moreover, when adjusting *RAI*-3'd2 for *RAI*-3'd1 we lost all association of the former with cancer, whereas *RAI*-3'd1 remained associated with cancer after adjustment for *RAI*-3'd2. This suggested that RAI-3'd2 was only indirectly associated with cancer, for instance through its linkage disequilibrium with *RAI*-3'd1.

We also performed haplotype trend regression on *RAI*-3'd1 and it's neighboring SNPs. One of the haplotypes, *RAI*-3'd1 *RAI-*3'd3 *RAI*-3'4, gave a lower overall p-value (p= 0.0002) than any we had seen (indicated as the singular value in Figure 3).

To better understand the nature of the association, we tabulated the frequencies of individual haplotypes made up of the 3 neighbouring markers centring on the position at 24 kb with maximal evidence of association with breast cancer (*RAI*-3'd1 *RAI*-3'd3 *RAI*-3'4, see above and Table 3). We also tabulated p-values associated with the individual frequencies. The same haplotypes in young and older cancer cases differed in frequency from the controls (*RAI*-3'd1^L^*RAI*-3'd3^S ^*RAI*-3'4^C ^protective; whereas all haplotypes containing *RAI*-3'd1^S ^seemed inductive).

**Table 3:** Frequency of haplotypes in breast cancer cases and controls and p-values for their association with cancer as determined by haplotype trend regression.

	Breast cancer cases			Breast cancer controls
		
Haplotype/Agegroup	Young^1)^	Old^2)^	All ages	All ages
*RAI*-3'd1^s ^*RAI*-3'd3^s ^*RAI*-3'4^c^	0.035^3 ^(0.37)^4^	0.054 (0.002)	0.051 (0.003)	0.022
*RAI*-3'd1^s ^*RAI*-3'd3^s ^*RAI*-3'4^t^	0.001 (-)	0.002 (0.12)	0.002 (0.09)	0.000
*RAI*-3'd1^s ^*RAI*-3'd3^l ^*RAI*-3'4^c^	0.008 (0.12)	0.006 (0.11)	0.006 (0.07)	0.001
*RAI*-3'd1^s ^*RAI*-3'3d^l ^*RAI*-3'4^t^	0.712 (0.02)	0.631 (0.17)	0.642 (0.06)	0.597
*RAI*-3'd1^l ^*RAI*-3'd3^s ^*RAI*-3'4^c^	0.244 (0.006)	0.297 (0.003)	0.290 (0.0005)	0.379
*RAI*-3'd1^l ^*RAI*-3'd3^s ^*RAI*-3'4^t^	0.000 (-)	0.005 (0.04)	0.005 (0.01)	0.000
*RAI*-3'd1^l ^*RAI*-3'd3^l ^*RAI*-3'4^c^	0.000 (-)	0.000 (0.61)	0.000 (0.11)	0.000
*RAI*-3'd1^l ^*RAI*-3'd3^l ^*RAI*-3'4^t^	0.000 (0.69)	0.003 (0.48)	0.003 (0.57)	0.001
*RAI *intron8-2^a ^*RAI *intron8-3^a^	0.259 (0.78)	0.209 (0.01)	0.216 (0.02)	0.271
*RAI *intron8-2^a ^*RAI *intron8-3^c^	0.000 (0.89)	0.007 (0.13)	0.006 (0.13)	0.000
*RAI *intron8-2^g ^*RAI *intron8-3^a^	0.69 (0.12)	0.701 (0.001)	0.699 (0.0007)	0.615
*RAI *intron8-2^g ^*RAI *intron8-3^c^	0.051 (0.05)	0.084 (0.11)	0.079 (0.04)	0.113

1) Breast cancer before age 55

2) Breast cancer at age 55 or older

3) Haplotype frequency

4) P-value for haplotype frequency in comparison to the haplotype frequency of controls, all ages.

Finally, as a preliminary check of our results we have tested *RAI*-3'd1 in a small independent sample of Danish women from Funen with breast cancers (Cold, unpublished). We found the persons that were *RAI*-3'd1^SS ^to have an increased risk of breast cancer between 50 and 60 years of age (Table 4). A similar, but non-significant tendency was present in subjects under age 50, whereas no association between alleles of *RAI*-3'd1 and breast cancer was present after age 60.

**Table 4:** Breast cancer risk in an independent small cohort of Danish Women in relation to *RAI*-3'd1.

Age at diagnosis		RAI-3'd1^SS^	RAI-3'd1^SL ^+ RAI-3'd1^LL^	OR^1 ^(95% CI^2^) (one-sided)	P-value^3 ^(one-sided)
< 50 years	cases	9	14	1.36 (0.51 -)	0.30
	controls	9	19		
50 – 60 years	cases	13	9	2.50 (1.02 -)	0.04
	controls	15	26		
>= 60 years	cases	30	44	0.89 (0.46 -)	0.88
	controls	32	38		

The table lists the number of persons in each group, the odds ratio and the lower 5 percent confidence limit for the distribution of persons, and the p-value for the odds ratio.

1) Odds Ratio for cases and controls combined with genotypes

2) One-sided lower 95% Confidence Limit for the OR

3) One-sided P-value for the OR

## Discussion

This study continues our studies of the association of breast cancer with one particular chromosomal region, 19q13.3, more specifically the gene *RAI *and its immediate surroundings. The present results improve the chromosomal resolution and suggest a possible effector, i.e. a causative variant. In addition, the haplotype analysis presented is the first evidence for association of *RAI *with breast cancer after age 55. It does so with p-values around 10^-3 ^at the same location as the analysis of younger persons, a testament to the efficiency of analyzing haplotypic associations rather than single SNPs. With this finding our results become relevant for a much larger group of cancer patients.

Most of our data come from a population-based follow-up study in Denmark with very limited dropout. Although this nested case-control study was originally individually matched, we have sometimes treated it as an unmatched case-control study in order to be able to use current computer programs. This opens the possibility of confounding from the matching parameters. For two reasons, however, we find such confounding highly unlikely. First, the observed effects are generally too strong for confounding to be a reasonable explanation. Second, confounders must correlate with the primary variables. We have no reason to believe that any of the match criteria correlated with the constitution on chromosome 19.

We used several ways to try to locate the effector(s). The overall conclusion was clear: One effector is located in the haplotype block between the markers *XPD *exon6 and *RAI *intron8-3. Details of the results varied: Methods handling one marker at a time indicated that the effector could be located anywhere from the 5' portion of *XPD *to the 5' portion of *RAI*, whereas analyses combining information from multiple markers indicated the presence of two effectors; one was located just 3' to the gene RAI, and another was located in the 5' portion of the gene (at 39 kb).

We resequenced the region of strongest association identified by haplotype trend regression. Here we discovered a new 5-base tandem repeat polymorphism, *RAI*-3'd1, that showed even stronger association with cancer (p = 0.0008). Bonferroni adjustment for the multiplicity of testing indicates that with an average correlation of 0.32 among a total of 68 polymorphisms, any p-value below 0.003 will make the whole set significant [17]). Thus, the result cannot be considered a result of the multiplicity of testing.

The polymorphism *RAI*-3'd1 may well be the effector. The haplotype data of Table 3 indirectly supports this view. All haplotypes in Table 3 containing *RAI*-3'd1^S ^were associated with increased risk of cancer, irrespective of the surrounding polymorphisms. We would not expect this result if *RAI*-3'd1 only was a correlate of the effector. However, mapping inside the haplotype block with the use of data from the present population is not very sensitive. For instance, the markers *XPD*-5'2 and *RAI *intron11, spaced 12 kb apart, were synonymous in 799 of 813 persons. Studies of this polymorphism in populations of different ethnicity could prove very useful, but functional assays will be necessary before a final conclusion can be reached.

It may be useful to compare aspects of the breast cancer risk caused by the *RAI *region with that caused by the *BRCA *genes. The *RAI *region is either recessive or suffers from strong haplo-insufficiency, and it roughly doubles the risk of breast cancer, thus the penetrance is low. The *BRCA *genes on the other hand are dominant, and they increase the breast cancer risk about 8 fold, i.e. penetrance is high. However, the risk allele of *RAI *is very common. In our study approximately 40 percent of the controls were homozygotes for the risk allele. A conservative estimate of the ethiological fraction suggests that at least 20 percent of breast cancers is caused by *RAI*-3'd1, and the number may be twice as high. Thus in terms of the number of cancers caused in the Danish population, *RAI *seems to exceed the BRCA genes in importance.

From Table 3 it is apparent that in the Danish population, and presumably in other Caucasian populations as well, the risk allele, *RAI*-3'd1^S^, occurred with different neighboring SNP alleles at reasonable frequencies. In contrast the protective allele *RAI*-3'd1^L ^almost exclusively occurred together with *RAI*-3'd3^S ^and *RAI*-3'4^C^. Further investigation has shown that this tendency was reproduced with a number of the surrounding SNPs. Generally, the less frequent haplotypes involving *RAI*-3'd1^S ^were approximately 10 fold more common than the less frequent haplotypes involving *RAI*-3'd1^L ^were. This was true for 12 out of 14 SNPs in the 23 kb interval from *XPD *exon6 to *RAI *intron8-1. The exceptions were *RAI*-e13 and *RAI*-3'7. In accordance, combined analysis of the 12 SNPs using HelixTree to form a 12 point haplotype indicated that 13 less frequent haplotypes involving *RAI*-3'd1^S ^constituted 23 percent of the chromosomes, corresponding to 56 percent of the major haplotype with *RAI*-3'd1^S^. In contrast, 7 less frequent alleles involving *RAI*-3'd1^L ^only constituted 2 percent of the chromosomes, corresponding to only 8 percent of the major haplotype with *RAI*-3'd1^L ^(results not shown). Thus, the haplotypes containing *RAI*-3'd1^S ^seem more diverse than their counterparts containing *RAI*-3'd1^L ^do. The simple interpretation of this phenomenon would be that *RAI*-3'd1^S ^is the ancestral allele in Caucasians.

There are at present no known polymorphisms affecting the amino acid sequence of the RAI protein. Presumably, *RAI*-3'd1 influences the expression of *RAI*. Our hypothesis is that *RAI*-3'd1 modulates a silencer or enhancer. The influence of *RAI*-3'd1 may not be limited to *RAI*. It is a curious fact that this short stretch of chromosome 19, comprising only 69 kb, contains two DNA repair genes and one apoptosis gene. One could imagine some sort of higher-order control common for this cluster of related genes. Studies of the expression of *XPD*, *RAI *and *ERCC1 *in lymphocytes from humans have indeed shown that their mRNA levels are tightly coordinated [18,19].

If a primary effect of *RAI*-3'd1 is modified *RAI *expression, one result should be modified apoptosis. RAI protein is an inhibitor of RelA, a subunit of the transcription factor NF-κB [20,21]. NF-κB has long been implicated in both cell proliferation and apoptosis. Modulation of NF-κB may well be part of a "crunch-time scenario", invoked when the cell has to muster its forces and make life-and-death decisions. A recent report suggested that the choice between cell survival and death is regulated by the relative activity of the two subunits encoded by *RelA *and *c-rel *[22]. By neutralising the *RelA *product, RAI protein would presumably shift this balance towards apoptosis. In accordance with this view, we have found that *RAI *mRNA increases dramatically when cells undergo apoptosis. We have also found that simultaneous transfection with siRNAs destabilizing *RAI *mRNA reduces apoptosis in non-transformed cells. Thus, we believe that *RAI *expression is instrumental for apoptosis [23]. However, RAI protein may also block p53 activity and thus have the opposite effect [24]. For instance, RAI is over-expressed in certain tumors and may in this situation block apoptosis. We have previously found that the expression of RAI is ca. 8-fold higher in both preneoplastic lesions such as adenomas and in tumour tissue such as adenocarcinomas compared to healthy tissue from the same person [25]. Furthermore, we have found that over-expression of RAI enhances the malignant character of oncogene-transformed cells (M. Laska et al, manuscript in preparation). Thus, RAI may alternatively accelerate or inhibit cell growth depending on the circumstances.

The previous reports, that have associated our region of interest with the risk of getting basal cell carcinoma, melanoma, lung cancer, glioma, breast cancer, bladder cancer, and possibly head and neck cancer [1-10,26,27], suggest that the effector may influence the risk of getting a wide variety of cancers of different etiology. This conclusion makes perfect sense if the functional elements modulate a downstream cellular response, such as apoptosis.

## Conclusion

We found the region around the RAI gene to be strongly associated with breast cancer, in particular during the early menopause. The marker *RAI*-3'd1 may be the cause for the extra risk for breast cancer. We think it may modulate an enhancer or silencer influencing *RAI *and possibly other genes.

## Methods

### Ethical compliance

This study was performed in compliance with the Helsinki declaration. The project was approved by the Science Ethical Committee of Copenhagen (j.nr: 01-345/93/(KF) 11-037/01/11-124/01). Written and verbal informed consent was obtained from all participants.

### Study groups

"Diet, Cancer and Health" is a Danish prospective follow-up study [28]. Individuals eligible for inclusion were born in Denmark, living in the Copenhagen or Aarhus areas, and at the time of inclusion had not been registered as having cancer (including non-melanoma skin cancer) in the Danish Cancer Registry. Invited to participate were 160 725 individuals, aged 50 to 64 years, of whom 57 053 were recruited. Among these, 542 were later registered with the Danish Cancer Register with a cancer diagnosed before the date of enrolment and were therefore excluded. At enrolment (1993–1997), detailed information was collected on diet, smoking habits, lifestyle, weight, height, children, medical treatment, and other socio-economic characteristics and environmental exposures. For women, information regarding hormone replacement therapy (HRT) and menopausal status was also recorded. Moreover, blood, urine, fat tissue, and other biological materials were sampled and stored at -150°C. Cohort members were identified by a unique identification number, allocated to every Danish citizen by the Central Population Registry. Cohort members were linked to the Central Population Registry for information on vital status and immigration. Information on cancer occurrence among cohort members was obtained through record linkage to the Danish Cancer Registry, which collects information on all inhabitants in Denmark who develop cancer [29]. Linkage was performed by use of the personal identification number.

The study sample for breast cancer included the 29 549 women recruited within "Diet, Health and Cancer". Eight women were excluded from the study because they did not fill in the lifestyle questionnaire. Because the present analysis aimed at the subgroup of women who were post-menopausal at study entry, we excluded 4844 supposedly pre-menopausal women, including 4798 women who had reported at least one menstruation no more than 12 months before entry and no use of HRT, nine women who gave a lifetime history of no menstruations, and 37 women who did not answer the questions about current or previous use of HRT, leaving 24 697 postmenopausal women.

Each cohort member was followed up for breast cancer occurrence from the date of entry, i.e. date of visit to the study centre until the date of diagnosis of any cancer (except for non-melanoma skin cancer), date of death, date of emigration, or 31 December 2000, whichever came first. A total of 434 women were diagnosed with incident breast cancer during the follow up period. Controls were selected among the remaining cohort participants using a nested case-control approach. One control was selected for each of the 434 cases. The control was cancer-free at the exact age at diagnosis of the case and was further matched on age at inclusion into the cohort (half-year intervals), certainty of postmenopausal status (known/probably postmenopausal), and use of HRT at inclusion into the cohort (current/former/newer). Of the 434 pairs (866 women; 434 cases and 434 controls, including two cases), 19 pairs were excluded due to lack of blood sample in either case or control, leaving 415 pairs for study. Additional pairs were excluded due to problems with the genotyping procedure in one or both members of the pair.(RAI-3'd1 105 pairs; RAI-3d'2 62 pairs).

### Resequencing of genomic DNA

Resequencing was performed on PCR fragments from DNA of persons selected for different haplotypes among the known markers in the region. 7 of the cases were further selected for having gotten breast cancer before age 55. The DNA sequences to be amplified and resequenced were obtained from databases. Repetitive sequences were determined and masked using RepeatMasker software. Primers were chosen using Primer3 software. PCR amplification was performed in a 10 μL volume and the product was purified with Centricon 100 (Ambion). Each sequencing reaction contained 2.5 μL of the purified PCR product, 6.5 μL of water, 2.0 μL of BigDye version 3 mix (Applied Biosystems), and 1.0 μL of 5× sequencing buffer, according to the protocol of the dye manufacturer. Electrophoresis and sequencing detection were performed using an ABI PRISM 3730 × l DNA Analyzer (Applied Biosystems).

### Typing of SNPs

Table 1 lists the polymorphisms used in this study, their nature, the numbers in the NCBI database dbSNP, the method and time of typing, and their position therein, and their relative position within the region of interest. Typing was performed on a Lightcycler, a Taqman and a Sequenom. Three polymorphism, RAI-3'd1, RAI-3'd3, and RAI-3'd2 were determined as length polymorphisms on an ABI3100 (Applied Biosystems, Nærum, Denmark). Information about the assay conditions can be found in the Additional File 1: SNP typing and SNP identification.

### Statistics

Data recording, calculations and tests of allele frequencies were performed in SPSS and Excel. Calculation of the relative risk and confidence intervals for the single polymorphisms was done using the procedure phrec in the program package SAS (SAS Institute, Cary, NC, USA). Simultaneous analysis of multiple SNPs employing haplotype trend regression [15] was performed with HelixTree (GoldenTree, Bozeman, MT, USA). Three programs were used for assigning haplotypes to individuals on the bases of genotype data: HelixTree, Arlequin [30] and Phase [31]. Arlequin like HelixTree is a maximum likelihood algorithm, whereas Phase also includes a penalty for each new haplotype that is brought into play. Furthermore, Arlequin includes missing values in its table of frequencies. To compensate for the latter, we normalised the values corresponding to fully defined haplotypes before including them in the analysis. All three were run under Windows 2000. HelixTree and Arlequin produced almost identical data. The data from Phase were also similar to those of the other programs. Helix Tree's values were used in the further calculations. The figure of the linkage disequilibrium among the controls (Figure 4) was also derived from HelixTree.

## Competing interests

The authors declare that they have no competing interests.

## Authors' contributions

BAN provided concepts and ideas, performed part of the data analysis and drafted the manuscript. UV contributed to the concepts and performed part of the marker analyses. AO performed part of the data analysis. MN, BH, ZB, ER, CK, MM, AK and ML established and performed most of the marker analyses. QZ performed the sequencing and part of the associated analysis. OR-N, SC, KO and AT established the cohorts. LB and ADB provided ideas and analyses.

## Pre-publication history

The pre-publication history for this paper can be accessed here:



## Supplementary Material

Additional file 1 **SNP typing and SNP identification**. The conditions for the SNP typing and the description of the SNPs found by sequencing are stored as a .pdf file in the Additional File 1. Click here for file
