# Supplementary material for: Linkage disequilibrium mapping of a breast cancer susceptibility locus near RAI/PPP1R13L/iASPP
Source: BMC Med Genet. 2008 Jun 27;9:56. doi: 10.1186/1471-2350-9-56 (PMC2474586; doi:10.1186/1471-2350-9-56)
Supplement: Additional file 1 — SNP typing and SNP identification. The conditions for the SNP typing and the description of the SNPs found by sequencing are stored as a .pdf file in the Additional File 1. [file 1471-2350-9-56-S1.doc]

**ADDITIONAL FILE 1**

*Typing of SNPs*. Typing was performed on a Lightcycler (Roche, Geneva, Switzerland), Sequenom(Sequenom, Inc, San Diego, USA), Taqman and ABI3100 (Aplied Biosystems, Nærum, Denmark). Table 6 lists the primers used for the PCR reactions. Table 7 lists the probes used for detection and typing. Table 8 lists techniques used for the PCR reaction, the type of reaction mixture, any modification of the reaction mixture used, and the temperature cycles. Taqman buffer denotes 1 ul MasterMix (Applied Biosystems, Nærum, Denmark), 600 – 900 nM primers (Tagc, Copenhagen, Denmark) 100 nM of each probe (Applied Biosystems) in 10 ul. Roche buffer denotes the 1 ul hybridisation probes mixture and 1 ul 25 mM MgCl_2_, produced by Roche Diagnostics (Geneva, Switzerland) in 10 ul; Homebrew denotes a mixture containing 0.25 ul Titanium Taq (BD Biosciences, Palo Alto, USA) 1 ul Titanium Taq buffer (ibid), 1.3 nmole dNTP in 10 ul. The latter mixtures also contained 10 pmole of each primer and 1 pmole of each probe. In both instruments, we used 45 cycles of amplification. Polymorphisms analysed on Taqman were scored on the basis of the relative reaction of the two probes. Polymorphisms analysed on Lightcycler were scored on the bases on the temperature dependency (“melting profile”) of the fluorescence. Because of a misidentification problem in the analysis of the two base deletion, *RAI*-3’3, we retyped it using nested PCR with one fluorescent second primer followed by length determination on an ABI3100. After correction of the misidentification, the two methods gave almost identical results. In case of a discrepancy, we used the result from the ABI3100. The primers, probes, and PCR programs used for the length analysis are included in tables 6, 7, 8.

*Results of Sequencing.* The polymorphisms found by sequencing 10 cases and 10 controls are listed in Table 9 with their position in the contig NT_011109.15, their position relative to XPD-exon23, and their surrounding sequence.

Table 6. Primers used for the assays of the polymorphisms

| Trivial name | Primers |
| --- | --- |
| *XPD-*exon23 | 5’ atg cac cag gaa ccg ttt atg g |
|  | 5’ tct gtt ctc tgc agg agg atc |
| *XPD-*exon10 | 5’ gat caa aga gac aga cga gc |
|  | 5’ gaa gcc cag gaa atg c |
| *XPD-*exon6 | 5’ gta cca gca tga cac cag cct |
|  | 5’ tcc ctc cct gag ccc tg |
| *XPD-*intron3-1 | 5’ aag gca gac aaa gga agg |
|  | 5’ gca agg aga agg aac agg |
| *XPD*-5’d1 | 5’ caa tca aaa aga aaa cat gg |
|  | 5’ tga gac gag gtg gag g |
| *XPD-*5’4 | 5’ atc aga cac atg gta gat gc |
|  | 5’ tct aac gga gaa tga cag g |
| *XPD*-5’d2 | 5’ tgc ctc acc cct gta atc c |
|  | 5’ gct tgt aat ccc agc tac tcg |
| *XPD*-5’2 | 5’ caa cac tca cac ccc aca g |
|  | 5’ aga tca cgc cac tgc act c |
| *XPD*-5’3 | 5’ ttg aca att gag caa aga gc |
|  | 5’ tgg gat tac aga cgt gag c |
| *RAI*-3’d2 | 5’ aga aca ata aag cag gga aag g |
|  | 5’ FAM-tt ggc aaa gca tgg tag c |
| *RAI*-3’7 | 5’ cca gtc caa aca ata tga tcc |
|  | 5’ agt gca gcc tca act tcc |
| *RAI-*3’d1 | 5’-aaa aaa ata gcc gag cat gg |
|  | 5’FAM-tt tgg act ggg taa gaa ttt cc |
| *RAI-*3’9 | 5’ gct ggg aat ata gac atg |
|  | 5’ aga cag ggt ctc act tc |
| *RAI*-3’d3 | 5’ cat gat tca ctg cac cca acc |
|  | 5’ ttt cac tct tgt tgc cca agc |
| *RAI-*3’10 | 5’ cag gtt caa gcg att ctc |
|  | 5’ gct agg ttg cac tat gat c |
| *RAI*-3’4 | 5’ ttt tca cac aag tcc aat cc |
|  | 5’ act gca acc tcc atc tcc |
| *RAI-*exon13-1 | 5’ ccc tgc ccc acc tct cc |
|  | 5’ agt caa ttt ctg tgc aaa cta ctt tta ttt |
| *RAI-*intron12-3 | 5’ cat gac gag acc ctg tct cta cta aa |
|  | 5’ cac ctc ccg gat tca agt ga |
| *RAI-*intron12-2 | 5’ gag gca aca gga aca aac c |
|  | 5’ cat tgg att gag cag aaa cc |
| *RAI-*intron11-1 | 5’ taa cat aaa gaa tca gga gga ggc |
|  | 5’ agt tgg ctc atc tgc ctc tt |
| *RAI-*intron8-1 | 5’ tgg cta aca cgg tga aac c |
|  | 5’ gga atc caa aga ttc tat gat gg |
| *RAI*-intron8-5 | 5’ gtc aga ttt ggt gac ttc aag |
|  | 5’ gag ccc tag agc att taa ag |
| *RAI-*intron8-2 | 5’ act cct gac ttc aaa tga tcc |
|  | 5’ tag ccc cca gtc acg ttc c |
| *RAI-*intron8-3 | 5’ aga agt cca aga gtt tgc agc |
|  | 5’ ttc tca gtc cca gaa tga acc |
| *RAI*-intro3-2 | 5’ cca ctt agg taa aca cct ctt |
|  | 5’ ctg caa tga gcc gag ata gaa |
| *RAI*-intron3-3 | 5’ cca ctt agg taa aca cct ctt |
|  | 5’ ctg caa tga gcc gag ata gaa |
| *RAI*-intron1-1 | 5’ atg ttg ggg aga ctg agg |
|  | 5’ ccg cat cta act tat tct gg |
| *RAI-*intron1-4 | 5’ gcc cct tta atc tct atg agc |
|  | 5’ cat cca tct aga ctt cca gag c |
| *RAI-*intron1-2 | 5’ aac tac ctc tgc aaa ccc agc |
|  | 5’ ttg gaa tgg agg gat tct acc |
| *RAI-*intron1-3 | 5’ agt tta ccc ctt cct ctc c |
|  | 5’ aac cca aca tgt cat taa cc |
| *RAI-*intron1-5 | 5’ ccc cac cag tct gag c |
|  | 5’ gga atg gag gga ttc tac c |
| *RAI-*5’1 | 5’ ggg aac agg tta gac gac g |
|  | 5’ atc agg aaa ggg gat agt gc |
| *ASE1*-exon1 | 5’ ggt ttt ctg ctc tgc aca cg |
|  | 5’ cct ttc tcc ttc cac caa cg |
| *ASE1*-exon3-1 | 5’-aca gag ccc aca gtg gag aca |
|  | 5’- cta gag gct cag tgt taa tct gtt cct |
| *ASE1*-exon3-2 | 5’ gga cag atg gca atg atg g |
|  | 5’ tct tct tct tgg tgg atg tgg |
| *ASE1*-exon3-d1 | 5’ HEX- cag ctc cca aaa aga aga cg |
|  | 5’ cgg ctt tcc tga ctc tgc |
| *ASE1*-exon3-6 | 5’ tct tga aag tac cct gat gac c |
|  | 5’ agt gac tga gcc aat tca gc |
| *ASE1-*exon3-4 | 5’ cga cag agc gag acc ctt ttc aaa |
|  | 5’ cac cct gcc aag tta ttt |
| *ASE1*-exon3-3 | 5’-acc atg gcg cct caa ca |
|  | 5'-gaa ttg gct cag tca ctg tgt ga |
| *ERCC1-*exon4 | 5’ ggc cct gtg gtt atc aag g |
|  | 5’ tct cat aga aca gtc cag aac act g |
| RAI-3’3 nested 1 | 5’ cat gat tca ctg cac cca acc |
|  | 5’ ttt cac tct tgt tgc cca agc |
| RAI-3’3 nested 2 | 5’ 6FAM-ctt gca cag tgg ctc atg c |
|  | 5’ tct tgt tgc cca agc tgg |

Table 7. Probes for the assays of the polymorphisms

| Trivial name | Probes |
| --- | --- |
| *XPD-*exon23 | 5’ FAM-ctc tat cct ctg cag cg-MGB |
|  | 5’ VIC-tat cct ctt gag cgt ct-MGB |
| *XPD-*exon10 | 5’ LC Red640- cgt gct gcc caa cga agt g –p |
|  | 5’ gga cgc cca cct ggc caa cc –fluoresceine |
| *XPD-*exon6 | 5’ FAM-ccc cac tgc cgc ttc tat gag gt-TAMRA |
|  | 5’ VIC-ccc cac tgc cga ttc tat gag gtt-TAMRA |
| *XPD-*intron3-1 | 5’ LC Red640- ccc tgc ccc cca act ttg ga -p |
|  | 5’ gcc tcc aat gaa cac aag ctc -fluoresceine |
| *XPD*-5’d1 | 5’ LC Red640- cct ggg ttc gat caa tac tca gac a –p |
|  | 5’ ctc gct atc ttg ctc aag ctg atc tcg aac –fluoresceine |
| *XPD-*5’4 | 5’ tag aga cag gtt ttc tcc at - fluoresceine |
|  | 5’ LC Red640- ttg gtc agg ctg gtc ttg - p |
| *XPD*-5’d2 | 5’ agt cac agc tca ctg cag cct c –fluoresceine |
|  | 5’ LC Red640- acc tct tgg gct caa gcg atc ctc –p |
| *XPD*-5’2 | 5’ LC Red640- aaa aaa aga ctt atc atg aca gga tgt ct –p |
|  | 5’ gca aga ctc cgt ccc aga aaa aga aaa –fluoresceine |
| *XPD*-5’3 | 5’ LC Red640- tcc tct ctc tcc ccc agc tca ttt tg -p |
|  | 5’ aac cca ccc tac tgc tct gat ctc -fluoresceine |
| *RAI*-3’7 | 5’ LC Red640- agg ctg gtc ttg aac tcc tgg gct taa g -p |
|  | 5’ ggt tcc gcc acg ttg cc -fluoresceine |
| *RAI-*3’9 | 5’ gtt tca agc gat tct cct g -fluoresceine |
|  | 5’ LC Red640- ctc agc ctc ccc agg a -p |
| *RAI*-3’d3 | 5’ LC Red640- tcg gct att ttt ttt ttt att ttt tta tt –p |
|  | 5’ att aca ggc acc cac cac cat g –fluoresceine |
| *RAI-*3’10 | 5’ ctg gtc tca aac tcc tga c -flouresceine |
|  | 5’ LC Red640- ttg tga tcc gcc tgc c -p |
| *RAI*-3’4 | 5’ LC Red640- cct gga caa cat agg gag acc ctg tgt -p |
|  | 5’ caa aca aac aaa aac ctc tgc ca -fluoresceine |
| *RAI-*exon13-1 | 5’ 6-FAM-tgc ctt cac aca gct ctg gtt taa tg – TAMRA  5’ VIC – tgc ctt cac aca gca ctg gtt taa tg – TAMRA |
| *RAI-*intron12-3 | 5’ FAM-tgg tgg tgc atg cct gta atc cc-BHQ |
|  | 5’ Yakima Yellow-tgg tgg tgc atg ccc gta atc-BHQ |
| *RAI-*intron12-2 | 5’ acc atg ttg gcc agg ctg gtt tt –fluoresceine |
|  | 5’ LC Red640- atc tac tga cct caa atg atc cac ct –p |
| *RAI-*intron11-1 | 5’ LC Red640- tgc aat ccg ccc gcc –p |
|  | 5’ cca ggc tgg ttt gga aat cct gag ctc –fluoresceine |
| *RAI-*intron8-1 | 5’ LC Red640- ctg aga tcg cac cac tgc ac –p |
|  | 5’ ggg agg cgg agc ttg cag tga –fluoresceine |
| *RAI*-intron8-5 | 5’ cct ggt ggc gca tgc ct –flouresceine |
|  | 5’ LC Red640- taa tcc cag cta ctc ggg agg ctc agg -p |
| *RAI-*intron8-2 | 5’ gcg cat gcc tgt aat tct gta –fluoresceine |
|  | 5’ LC Red640- cag gac gag cca cag aca aaa ctc c –p |
| *RAI-*intron8-3 | 5’ LC Red640- tgc aat gag gct cct ggc c -p |
|  | 5’ act aca ttt ccc agc atc cca -fluoresceine |
| *RAI-*intron3-2 | 5’ cct ccc tcc ctc cct gc –fluoresceine |
|  | 5’ LC Red640- tgc ttg ctt tct ctc tct -p |
| *RAI*-intron3-3 | 5’ tcc ctg ctt gct tgc ttt ctc t -fluoresceine |
|  | 5’ LCRed 640- tct ctc ttt ctt tct ttc ttt c -p |
| *RAI*-intron1-1 | 5’ tgt tca tcc aaa tga gcc gc –fluoresceine |
|  | 5’ LC Red640- agc ctg aac agg ttc tgt tcc ttc gac tt –p |
| *RAI-*intron1-4 | 5’ aac ccc tca gct aaa gag cct att –fluoresceine |
|  | 5’ LC Red640- ttg gaa agt tct gag tcc aa -p |
| *RAI-*intron1-2 | 5’ LC Red640- caa gct gct atc tcg acc gat ctt –p |
|  | 5’ ggg tga cca ccc tgc cag cc –fluoresceine |
| *RAI-*intron1-3 | 5’ aga act acc cat gca aac c –fluoresceine |
|  | 5’ LC Red 640- agc tgt ttc cca ccc cat a -p |
| *RAI-*intron1-5 | 5’ cga acc cct ata cta ccc a –fluoresceine |
|  | 5’ LC Red 640- aga ctc ggc ttc cta gag cc -p |
| *RAI-*5’1 | 5’ cgt agc cca aga cga tc -fluoeresceine |
|  | 5’ LC Red640- cat ttg gat tca ccc aga gtc c -p |
| *ASE1*-exon1 | 5’ LC Red640- cgg gct aca ggg tta cct gag –p |
|  | 5’ tct gca acc tgg tgc gag cag c –fluoresceine |
| *ASE1*-exon3-1 | 5’ FAM-aaa ggg a**a**a gaa acc t-MGB |
|  | 5’ VIC-cca aag gga **c**ag aaa-MGB |
| *ASE1*-exon3-2 | 5’ LC Red640- tga ggc tcc gct cct tct gg –p |
|  | 5’ agc tgc cag agc tgc ctg ggc –fluoresceine |
| *ASE1*-exon3-6 | 5’ cag act aca cag gct gct gct -fluoresceine |
|  | 5’ LC Red640- ctg ctg ctt ccg ctt -p |
| *ASE1-*exon3-4 | 5’ att atg tta agt gaa ata agc cag gca -fluoresceine |
|  | 5’ LC Red640- aga aag aca aac att gca tgt t -p |
| *ASE1*-exon3-3 | 5’ VIC-aca gca a**a**a tgc cac agt-MGB |
|  | 5’ FAM-aca gca a**g**a tgc cac ag-MGB |
| *ERCC-*exon4 | 5’ cgc aac gtg ccc tgg gaa t -fluorescein |
|  | 5’ LC Red640- tgg cga cgt aat tcc cga cta tgt gct g –p |

Table 8. PCR-regimen for the assays the polymorphisms

| Trivial name | Technique | Buffer | Buffer modification | Denaturation | Annealing | Elongation |
| --- | --- | --- | --- | --- | --- | --- |
| *XPD-*exon23 | Taqman | Mastermix | - | 15 sec 94 °C |  | 60 sec 60 ºC |
| *XPD-*exon10 | Lightcycler | Roche | 5% DMSO | 10 sec 95 °C | 15 sec 53 ºC | 30 sec 72 ºC |
| *XPD-*exon6 | Taqman | Mastermix | - | 15 sec 94 °C |  | 60 sec 63 ºC |
| *XPD-*intron3-1 | Lightcycler | Homebrew | - | 2 sec 95 °C | 15 sec 55°C | 30 sec 72 ºC |
| *XPD*-5’d1 | Lightcycler | Homebrew | 2x Buffer | 10 sec 95 °C | 15 sec 60 ºC | 30 sec 72 ºC |
| *XPD-*5’4 | Lightcycler | Homebrew | 2x dNTP | 2 sec 95 °C | 15 sec 62 ºC | 30 sec 72 ºC |
| *XPD*-5’d2 | Lightcycler | Homebrew | 5% DMSO | 10 sec 95 °C | 15 sec 66 ºC | 30 sec 72 ºC |
| *XPD*-5’2 | Lightcycler | Homebrew | 2x dNTP + 2x Buffer | 10 sec 95 °C | 15 sec 64 ºC | 30 sec 72 ºC |
| *XPD*-5’3 | Lightcycler | Homebrew | - | 2 sec 95 °C | 15 sec 71 ºC | 30 sec 72 ºC |
| *RAI-*3’d2 | ABI3100 | Homebrew |  | 30 sec 94 °C | 30 sec 60 °C | 36 sec 72 °C |
| *RAI*-3’7 | Lightcycler | Homebrew | - | 2 sec 95 °C | 15 sec 67 ºC | 30 sec 72 ºC |
| *RAI-*3’d1 | ABI3100 | Homebrew |  | 2 sec 95 °C | 15 sec 76 °C | 30 sec 72 °C |
| *RAI-*3’9 | Lightcycler | Homebrew | - | 2 sec 95 °C | 15 sec 65 ºC | 40 sec 72 ºC |
| *RAI*-3’3 | Lightcycler | Homebrew | 2x dNTP + 2x Buffer | 2 sec 95 °C | 15 sec 70 ºC | 30 sec 72 ºC |
| *RAI-*3’10 | Lightcycler | Homebrew | 2x dNTP | 2 sec 95 °C | 15 sec 67 ºC | 20 sec 72 ºC |
| *RAI*-3’4 | Lightcycler | Homebrew | - | 2 sec 95 °C | 15 sec 67 ºC | 30 sec 72 ºC |
| *RAI-*exon13-1 | Taqman | Mastermix | - | 15 sec 94 C |  | 60 sec 60 ºC |
| *RAI-*intron12-3 | Taqman | Mastermix | - | 15 sec 94 C |  | 60 sec 63 ºC |
| *RAI-*intron12-2 | Lightcycler | Homebrew | - | 2 sec 95 °C | 15 sec 60 °C | 40 sec 72 °C |
| *RAI-*intron11-1 | Lightcycler | Homebrew | 2x dNTP | 2 sec 95 °C | 15 sec 63 °C | 30 sec 72 °C |
| *RAI-*intron8-1 | Lightcycler | Homebrew | 2x Buffer | 0 sec 95 °C | 10 sec 57 ºC | 15 sec 72 ºC |
| Nr51 | Lightcycler | Homebrew | - | 2 sec 95 °C | 15 sec 62 °C | 30 sec 72 °C |
| *RAI-*intron8-2 | Lightcycler | Homebrew | - | 2 sec 95 °C | 15 sec 66 °C | 30 sec 72 ºC |
| *RAI-*intron8-3 | Lightcycler | Homebrew | - | 2 sec 95 °C | 15 sec 65 ºC | 30 sec 72 ºC |
| *RAI*-intro3-2 | Lightcycler | Roche | - | 2 sec 95 °C | 15 sec 61 ºC | 30 sec 72 ºC |
| *RAI*-intron3-3 | Lightcycler | Roche | - | 2 sec 95 °C | 15 sec 61 ºC | 30 sec 72 ºC |
| *RAI*-intron1-1 | Lightcycler | Homebrew | 1M Betain | 2 sec 95 °C | 15 sec 63 ºC | 30 sec 72 ºC |
| *RAI-*intron1-4 | Lightcycler | Homebrew | - | 2 sec 95 °C | 15 sec 66 ºC | 30 sec 72 ºC |
| *RAI-*intron1-2 | Lightcycler | Homebrew | 5% DMSO + 1½x Primer | 2 sec 95 °C | 15 sec 63 ºC | 30 sec 72 ºC |
| *RAI-*intron1-3 | Lightcycler | Homebrew | - | 2 sec 95 °C | 15 sec 63 ºC | 30 sec 72 ºC |
| *RAI-*intron1-5 | Lightcycler | Homebrew |  | 2 sec 95 °C | 15 sec 64 ºC | 12 sec 72 ºC |
| *RAI-*5’1 | Lightcycler | Homebrew | - | 2 sec 95 °C | 15 sec 66 ºC | 30 sec 72 ºC |
| *ASE1-*exon1 | Lightcycler | Homebrew | - | 2 sec 95 °C | 15 sec 60 ºC | 30 sec 72 ºC |
| *ERCC1*-3’2 | Taqman | Mastermix |  | 15 sec 94 °C |  | 60 sec 60 ºC |
| *ASE1-*exon 3-2 | Lightcycler | Homebrew | - | 2 sec 95 °C | 15 sec 62 ºC | 30 sec 72 ºC |
| *ASE-*exon3-d1 | ABI 3100 | Homebrew | - | 30 sec 94 °C | 30 sec 64 ºC | 27 sec 72 ºC |
| *ASE-*exon3-6 | Lightcycler | Homebrew | - | 2 sec 95 °C | 15 sec 66 ºC | 30 sec 72 ºC |
| *ASE1-*exon 3-4 | Lughtcycler | Homebrew | - | 2 sec 95 °C | 15 sec 60 ºC | 30 sec 72 ºC |
| *ERCC1-*3’3 | Taqman | Mastermix |  | 15 sec 94 °C |  | 60 sec 62 ºC |
| *ERCC1*-exon4 | Lightcycler | Homebrew | 5% DMSO | 0 sec 95 °C | 15 sec 57 ºC | 25 sec 72 ºC |
| *RAI*-3’d3 nested 1 | ABI 3100 | Homebrew | - | 30 sec 94 °C | 30 sec 64 °C | 30 sec 72 °C |
| *RAI*-3’d3 nested 2 | ABI 3100 | Homebrew | - | 30 sec 94 °C | 30 sec 62 °C | 30 sec 72 °C |

Table 9. Polymorphisms found by sequencing, including their number in dbSNP (if any), their position in NT_011109.15 and their surrounding sequence.

| **dbSNP** | **contig nt_011109** | **Sequence** |
| --- | --- | --- |
| rs1799793 | 18135477 | AACCCCGTGC TGCCC [A/G] ACGAAGTGCT GCAGGGTGAG |
|  | 18136224 | GACTCACAGC [A/T] AGCAAACAGACA |
|  | 18135477 | CAGACAGGAGAC [A/G] GGCGGGCAGCCC |
| rs238406 | 18136527 | CCTGCCCTCCAGTAACCTCATAGAA[G/T]CGGCAGTGGGGCAGGCTGGTGTCAT |
| rs238407 | 18136696 | GGAAACCCAACCACTCTTCAAACCC[A/T]GGTCTCCACACCTCCCAGCTGGCTG |
|  | 18136865 | GAGGCTGAGGTG [A/G] GCAGATCACGAGG |
|  | 18136867 | GAGGCTGAGGTGGG [C/G] AGATCACGAGG |
| rs3916809 | 18137426 | GGCCGCAGCTACTCGGGAGGCTGAG[C/T]GGGGGAAGGATCACTTGAGCCTGGG |
|  | 18137630 | GCCAAAACTAATATTTT [-/ATTTT] ATTTTTTGAGACGGA |
|  | 18137808 | TAGTAGAGACA [A/G] GGTTTCTCCA |
| rs3916808 | 18138367 | ATTACAGGCACCCACCACCACGCCC[G/T]GCTAGTTTTTTGTATTTTTAGTAGA |
|  | 18138512 | AAACTCCATTTC [A/G] AAAATAATAATAATAAAT |
|  | 18138675 | TTAGCCGGGTG [C/T] AGTGGGGCACCTG |
| rs10421181 | 18138844 | AACAAAACAAAAATATATATATATA[C/T]ACACACACACACACACACACACACA |
| rs8109829 | 18138876 | ACACACACACACACACACACACACA[C/T]ATATAAAAACATATATACACACACA |
|  | 18139824 | CCATAAATAC [C/T] GGGCTAGTAAGTTT |
| rs1799783 | 18140254 | GGGCAGGGGTTTGTGCCTCCAATGA[A/G]CACAAGCTCCCCCTGCCCCCCAACT |
| rs3810366 | 18142160 | ACCTGCCCGTCAATCCGCTAGGGCA[C/G]AGCCAATCGGGATACTGCGCGTGCG |
| rs3916791 | 18142345 | AACTCCTGGGTTCGATCAATACTCA[-/GACA]ATCTTGGCAGGCGCAGGAGGACCAA |
| rs3916788 | 18142630 | ATTTTGTATCTTTAGTAGAGACAGG[G/T]TTTCTCCATGTTGGTCAGGCTGGTC |
|  | 18143581 | CAGGAGTGAAA [A/C] CCTATCTCAAA |
| rs2097215 | 18144005 | CAGTTGACAGTAGACATCCTGTCAT[A/G]ATAAGTCTTTTTTTTTTTTCTTTTT |
| rs11878944 | 18145185 | ACAGACACACACACCGACATGGCCT[C/T]GCTCTCGCTCTCTCTCACACACACA |
|  | 18145748 | TCCTAGACACACACA [-/CACACGCACGCACGCACGCACA] CACACACACGCACGCA |
|  | 18146006 | CCTGACCAACAC [A{G] GTGAAACCTCAT |
| rs10411647 | 18146083 | CATGCCTGTAATCCCAACTACTCAG[A/G]AGGCTGAAGCAGGAGAATCGCTTGA |
|  | 18146268 | GGCCGAGGCGG [G/T] CGGATCACTTGAGGT |
| rs7252567 | 18146823 | TTTTAGTAGAGACATGGTTCCGCCA[C/T]GTTGCCCAGGCTGGTCTTGAACTCC |
|  | 18147012 | ATTTTATTTT[ATTTT]_n_ATTATTTTTGAGATGGATTTTCA |
| rs2377329 | 18147126 | CTGGGGAGGCTGAGGCAGGAGAATC[A/G]CTTGAAACCGGGAGGCGGAGGTTGT |
| rs3047560 | 18147192 | ACTAAAAATAAAAAAATAAAAAAAA[-/AA]ATAGCCGAGCATGGTGGTGGGTGCC |
|  | 18146233 | GATTGTCATGT [G/T] ACATCAGCCAATA |
| rs10422489 | 18147886 | AAAAAACTAAAGTGGGGTTTGCGGG[G/T]AGTGGGAGGGCCCTTCCTGCTAGGT |
| rs10426701 | 18148193 | CAGGCGGATCACAAGGTCAGGAGTT[C/T]GAGACCAGCCTGGCCAACACAGTGA |
|  | 18149120 | CACAGTGAAAC [C/T] CCATCTCTACTAAAAAT |
|  | 18149154 | AAATTAGCCGG [A/G] CGCCATGGCGGGA |
| rs4544343 | 18150199 | CCCTATGTTGTCCAAGCTGGCAGAG[A/G]TTTTTGTTTGTTTGTTTGAGAGGGA |
|  | 18150815 | GGCCAACATG [C/G] TGAAACCCCGTCTCT |
| rs8101662 | 18150911 | CTCGGGAGGCTGAGGCAGGAGAATC[A/G]CTTGAACTCAGGAGGCAGAGGTTGC |
|  | 18151158 | AAAGTTTCTCTATT [G/T] TGTTTATAAACATT |
| rs6966 | 18151180 | CCCCAATTAAGTGCCTTCACACAGC[A/T]CTGGTTTAATGTTTATAAACAAAAT |
|  | 18151772 | ATGCAAATCCGCTG [C/T] CTGTCTCTATCCTCCC |
|  | 18152034 | GGGTCACAC [C/G] TATAATCCCAGCAGTTT |
| rs10417235 | 18152171 | TTAGCTGGGCGTGGTGGTGCATGCC[C/T]GTAATCCCAGCTACTTGGGAGGCTG |
| rs12976252 | 18152764 | TGTTGGCCAGGCTGGTCTAGAACTC[C/T]TGACCTCAGGAGATCCGCCCGCCTT |
| rs8112723 | 18153497 | GGCAGGTGGATC [A/T] TTTGAGGTCAGTAGA |
|  | 18154712 | CTGACCTTGTGATC [C/T] GCCCACATTGGCC |
|  | 18154796 | TTTTTTGAG [A/C] TGGAATTTCGCTCTTGTCGC |
|  | 18155408 | CCCGGCTAAATT [G/T] TTTTATATTTTTAATAG |
| rs2017104 | 18155483 | GCACTTTGGGAGGCTCGAGGCGGGC[A/G]GATTGCATGAGCTCAGGATTTCCAA |
|  | 18155871 | AAAAAGTTAGCGGG [A/C] CGTGGGGCCCTTGCCTGTAA |
| rs7257687 | 18156146 | AGTTCGGGACCAGCCTGGGCAACAC[C/T]GCAAAACCCCTTCTGTACTAAAAAT |
|  | 18156162 | AAAACCCCTTCTG [C/T] ACTAAAAATACA |
| rs1970764 | 18159091 | GGAGCTTGCAGTGAGCTGAGATCGC[A/G]CCACTGCACTCCAGCCTGGGCGACA |
|  | 18159263 | GTTCTCCTGACCTC [A/G] TGATCCGCCCACCTGGAAAAAAT |
|  | 18160363 | TTACAGGCATGC [A/G] CCACCAGGCCCAGCTAA |
|  | 18160936 | CCAATGGTGACA [A/C] CAGTAAGAGCAGTTAACAGTT |
|  | 18160937 | CCAATGGTGACAA [C/G]AGTAAGAGCAGTTAACAGTT |
| rs10419090 | 18161433 | TACAGGCGCCCGCCACCACCCCCAG[A/C]TAATTTTTGTATTTTTAGTAGAGAC |
|  | 18161694 | GCCTCCTGA [G/T] TAGCTGGGATTG |
|  | 18161841 | TAAATAGCTAGA [C/T] GACCTTGGCGCCA |
|  | 18161896 | AAAATAATAATAATAATATTAA [C/T] CCCTGACCAAAACTAC |
| rs6509210 | 18162206 | TCGTCCTGCTACAGAATTACAGGCA[C/T]GCGCCACCGCTCCGGGCTAATTTTT |
|  | 18162309 | GATCCACCCAC [C/T] TCGGCCTCCCAAAGTGC |
|  | 18162356 | GAGCCACCGCGCCC [A/G] GCCGAGACTCACTATTT |
| rs12986272 | 18162599 | TAAAGCGGGAGGATGGCTTGAACCT[A/G]GGAGGCGGAGGTTGCAGTGAGCCGA |
|  | 18162620 | GGTTGCAGTGA [?/G] CCGAGGTCAAGCCACT |
|  | 18162903 | GGAGCAGAGAAC [A/C]TCTCTAGTGGCCA |
|  | 18162970 | CCTAAAGACTAC [A/C] TTTCCCAGCATCCC |
|  | 18162986 | TTTCCCAGCATCCCA [C/T] TGCAAATGAGGCTCCTGG |
|  | 18163200 | TCCTGACTCCAGTG [A/C] GGTGCCTACAGT |
|  | 18165052 | CCTGGGCAAGAA [C/G] AGTGAAACTCCATCTT |
| rs4803814 | 18168944 | CCCTCCCTGCTTGCTTGCTTTCTCT[C/T]TCTCTCTTTCTTTCTTTCTTTCTTT |
| rs4803815 | 18168948 | CCCTGCTTGCTTGCTTTCTCTCTCT[C/T]TCTTTCTTTCTTTCTTTCTTTCTTT |
|  | 18169725 | CCAGCCCCGCCC [C/T] CTACTCCAGCTTACACTGG |
| rs11673653 | 18171036 | AACGGCGTGAACCCGGGAGGTGGAG[C/T]TTGAACTGAGCGGAGATCGCGCCAC |
|  | 18171119 | AAAACAAACAAATAAATAAA [-/TAAG] TATGTTTAAAACAACAAC |
| rs1041973 | 18171624 | CAACTTCTGAAGTTTCTACCAGCTG[A/C]AGTTGCTGATTCTGGTATTTATACC |
| rs10402584 | 18171746 | CAAGAGGAACAGGGAGGTGGCCAGG[G/T]GTGGAGGGGCAGCTGTGGTCACTGG |
| rs10402393 | 18171815 | GGCCACTAGGAACCGGTCAGGCCAG[C/T]ACCATCCCTATCCCCATGCTAGCCA |
| rs4572514 | 18171984 | AGAACCTGTTCAGGCTGGCGGCTCA[C/T]TTGGATGAACAGGGAGTGTGTGACC |
|  | 18173106 | TATTGTTGGAAAGTT[C/T] TGA[g/c]TCCAAGATTCTATCTTT |
|  | 18173110 | TATTGTTGGAAAGTT[c/t] TGA[G/C]TCCAAGATTCTATCTTT |
|  | 18155974 | TCGGGGGAGAGCCGGGAC[A/G]CAGCCTCCGGAGGGA |
|  | 18176078 | GCTCAGAGACCTAGAAGATCGGTCGAGA[C/T]AGCAGCTTGAGG |
|  | 18176091 | AGCAGCTTGAGGCTGG[C/T]AGGGTGGTCACCCATTCCAC |
|  | 18176132 | CCACCTTGAGCCCCACCAGT[C/G] TGAGCCTCTCATTTCTGACCAAGA |
| rs4803817 | 18176178 | CTCGGGGATTCGAACCCCTAT[A/G]CTACCCAAAGACTCG |
| rs10412761 | 16176679 | GGGACAGGACGTAGCCC[A/G]AGACGATCCCATTTGGATTCACCCA |
|  | 18177268 | CACTGGCCAGGAATGCAGTCGGGTCAC[C/T]CTGTCTAGCCACCGTCT |
| rs967591 | 18178152 | GTGCGAGCAGCCCGGGCTACAGGGTT[A/G]CCTGAGGTGTGGGTCCCA |
|  | 18178221 | GGTGCGGGTTGACGGGGTGCG[G/T]AGGGTGCGTTGGTGGAAGGAGAAA |
|  | 18178589 | CCTTCTGCTGCAGATGCTGCTCGG[C/T] TCTCTTGTCCCC |
|  | 18178623 | CGCGAAGCCCCCAGCCTCA[C/G] AGTCCCCTCGTTTCTCCTTGGAG |
|  | 18178890 | AGAAAGCAGGGGGAGAAACCCAC[A/G] GCCCTTTGTTAGTATTTCTA |
|  | 18179361 | TGGCCAACATAGTGAAATTGT[C/T]T[c/t]TACTAAAAATACAAAAATTGGC |
|  | 18179363 | TGGCCAACATAGTGAAATTGT[c/t]T[C/T]TACTAAAAATACAAAAATTGGC |
|  | 18179400 | CTAAAAATACAAAAATTGGCTGGG[C/G] GTGGTG-GCAGGTGCCTGTA |
|  | 18179441 | GTAATCCCAGCTACTTGAGAGG[C/G] TGAGGCAGGAGAATCGC |
|  | 18179525 | CAGCCTAGGCAACAGAGCAAGACTCT[C/T] TCAAAAAAAAACAAA |
|  | 18179645 | GGCATGTGCCTCTCTCTGGCTCCCA[A/G] ATCGTCAAGGGCAAATTG |
|  | 18179715 | CCTCAGCAGCTGTCCCCAAGCTGGA[A/G] AAGCGACCCTGCTGGCC |
| rs762652 | 18180561 | GCGCCTCAACAGCCAGAAGGAGCG[A/G] AGCCTCAGGCCCA |
| rs2336219 | 18180624 | GAAGAAAGAAAAACAGCAA[A/G] ATGCCACAGTGGAGCCAGAGAC |
| rs3212987 | 18180725 | CAGCTCCCACATCCACCAAGAAGAAGAAGAAGAA[-/GAA] AGAGAGAGGTCACACAGT |
| rs3212986 | 18180954 | CAGGCCGGGACAAGAAGCGGAAG[A/C] AGCAGCAGCAGCAGCCTGTGTAGTC |
